# Pediatric Gastrostomy Tube Placement: Lessons Learned from High-performing Institutions through Structured Interviews

**DOI:** 10.1097/pq9.0000000000000016

**Published:** 2017-02-23

**Authors:** Loren Berman, Carla Hronek, Mehul V. Raval, Marybeth L. Browne, Charles L. Snyder, Kurt F. Heiss, Shawn J. Rangel, Adam B. Goldin, David H. Rothstein

**Affiliations:** From the * Nemours-A.I. duPont Hospital for Children, Wilmington, Dle.; † Sidney Kimmel College of Medicine, Philadelphia, Pa.; ‡ Children’s Hospital Association, Overland Park, Kans.; § Emory University, Children’s Healthcare of Atlanta, Atlanta, Ga.; ¶ Lehigh Valley Children’s Hospital, Morsani College of Medicine, University of South Florida, Allentown, Pa.; ‖ Children’s Mercy Hospital, Kansas City, Mo.; ** Boston Children’s Hospital, Harvard Medical School, Boston, Mass.; †† Department of Pediatric General and Thoracic Surgery, Seattle Children’s Hospital, Seattle, Wash.; ‡‡ University of Washington School of Medicine, Seattle, Wash.; §§ Department of Pediatric Surgery, Women and Children’s Hospital of Buffalo, Buffalo, N.Y.; and ¶¶ Department of Surgery, University at Buffalo Jacobs School of Medicine and Biomedical Sciences, Buffalo, N.Y.

## Abstract

**Introduction:**

Gastrostomy tube (GT) placement is one of the most common operations performed in children, and it is plagued by high complication rates. Previous studies have shown variation in readmission and emergency room visit rates across different children’s hospitals, with both low and high outliers. There is an opportunity to learn how to optimize outcomes by identifying practices at high-performing institutions.

**Methods:**

Surgeons and nurses routinely involved in GT care at 8 high-performing pediatric centers were identified. We conducted structured interviews focusing on the approach to GT education, technical aspects of GT placement, and postoperative management. Summary statistics were performed on quantitative data, and the open-ended responses were analyzed by 2 independent reviewers using content analysis.

**Results:**

Several common practices among high-performing centers were identified (standardized approach to education, availability by phone and in clinic to manage GT-related issues, and empowering families to feel confident with troubleshooting and dealing with GT problems). There was substantial variation in operative technique and postoperative care. The participants expressed that technical aspects of operative placement and postoperative management of feedings and common complications are not as important as education, availability, and empowerment in optimizing outcomes.

**Conclusions:**

We have identified common themes among pediatric centers with favorable outcomes after GT placement. Identifying which components of GT care are associated with optimal outcomes is critical to our understanding of current practice and may help identify opportunities to improve care quality.

## INTRODUCTION

Gastrostomy tube (GT) placement is one of the most common surgical procedures performed in the pediatric population.^1^ Recent reports highlighting the high rate of unanticipated emergency department (ED) visits and hospital readmissions after GT placement have sought to identify factors that may be predictive of such outcomes and strategies for decreasing unnecessary postoperative hospital resource utilization.^2–4^ Unanticipated 30- and 90-day ED visits and hospital readmissions after GT placement in patients younger than 18 years of age occurred at a high rate in a recent report using the Pediatric Health Information System (PHIS).^2^ Wide interhospital variability was found in revisit and readmission rates.

Pediatric readmissions in general have also been the subject of multiple studies, in both surgical and medical disciplines,^5–7^ and have drawn particular attention in recent years due to the mandates and financial penalties imposed on hospitals under the Affordable Care Act for excessively high readmission rates.^8^ This study uses qualitative and quantitative techniques in the form of structured interviews with medical providers at hospitals with particularly low revisit and readmission rates^2^ to identify potentially generalizable strategies to improve outcomes after pediatric GT placement.

## METHODS

### Study Population

PHIS data were previously analyzed with respect to outcomes after GT placement,^2^ and high-performing institutions were identified. There were a total of 38 hospitals included in the analysis. Average annual GT placement volume was 159 ± 61 (range, 28–297). The mean GT placement rate per 1,000 patient discharges was 10.9 ± 2.1 (range, 2.4–16.7). The mean percentage of GT placements that were scheduled was 23.5% ± 11.1% (range, 5.4–55.5%). The rates of readmission and ED visits and 95% confidence intervals were generated from a risk adjustment model. If a hospital’s 95% confidence interval did not include the overall mean, the hospital was considered a statistical outlier. An institution was designated as a high performer if it was found to be a low outlier in at least 2 of the following 4 categories: 30-day postoperative readmissions, 90-day postoperative readmissions, 30-day postoperative ED visits, and 90-day postoperative ED visits, and was not found to be a high outlier in any of these categories (Table 1). Of note, ED visits and readmissions were only counted if the reason for presentation was related to the GT. Interviews were conducted with surgeons and nurses at a total of 8 institutions. A surgeon was identified at each institution and asked to participate or name an alternative surgeon involved in care of GT patients and that person was then contacted and interviewed. The surgeon was then asked to identify the person at his or her hospital who is most involved with GT teaching, postoperative care, and long-term care, and this individual [a nurse or nurse practitioner (NP)] was also interviewed. At the time of these interviews, participants were asked about their partners’ practices and whether there were other contacts in their institution who would offer additional insights, and these individuals were also interviewed.

**Table 1.**
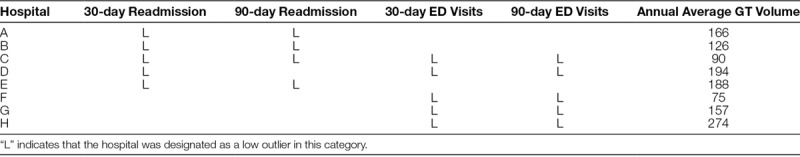
Summary of Outlier Status Among High-performing Hospitals

### Data Collection

Structured interviews were conducted with both closed and open-ended questions to describe the approach to GT care at 8 high-performing PHIS hospitals. Interviews were conducted by 1 of 5 surgeon interviewers. Notes were transcribed during the interview using a third-party note-taker when possible. Interview questions were divided into 3 categories: education, operative technique, and GT care. All 3 categories of questions were asked of the surgeon, and only the questions pertaining to GT education and GT care were asked of the nurses and NPs. The interview questions were designed to collect both quantitative and qualitative information. For example, the question was asked: “Is the stomach routinely sutured to the abdominal wall during GT placement?” with follow-up questions including “How is this accomplished?” and “Do you think this impacts outcomes?” (see Appendix for full interview guide). Using this interview technique, we were able to not only describe the approaches to education, technique, and care of GT patients at high performing institutions but also understand the rationale behind which approach and features were thought to be linked to good outcomes. With the open-ended questions, we intended to describe novel approaches and iteratively define best practices.

Although multiple surgeons conducted interviews, we attempted to maximize interrater reliability by standardizing our questions, discussing the interview style and format on group conference calls before the study, and having a third-party interview observer on as many calls as possible.

### Data Analysis

Two independent reviewers categorized the interview responses into qualitative and quantitative categories. Summary statistics were performed for the quantitative data. Qualitative responses were analyzed using the constant comparative method, a systematic data-coding and analysis procedure.^9,10^ This method involves the categorization of specific quotes from participants with the use of codes developed iteratively to reflect the data. We focused our analysis on those aspects of the qualitative data that would enhance our interpretation of the quantitative findings and define best practices for GT education, placement, and long-term care.^11^ Our findings were shared with participants once data analysis was complete to confirm that their perspectives were accurately represented.

## RESULTS

A total of 18 people were interviewed across the 8 institutions. Each site had a surgeon and nurse/NP interviewee, and 2 sites had an additional nurse/NP identified as significantly involved in GT care/education.

### GT Education

Surgeon and nurse participants at all 8 sites identified the nurse as the primary provider of education both before and after surgery (Table 2). An NP explained that education is provided by “the whole team” with a “post-op visit from the NP.” A surgeon role was identified formally in only 2 sites. One surgeon described both inpatient and outpatient education being a joint effort of the “surgeon and nurses.” Six sites specifically noted the involvement of the NP, whereas 2 sites referred to specialty or clinic nurses. Nursing staff who work on the patient care units where GT patients are admitted postoperatively were also noted as important educational providers.

**Table 2.**
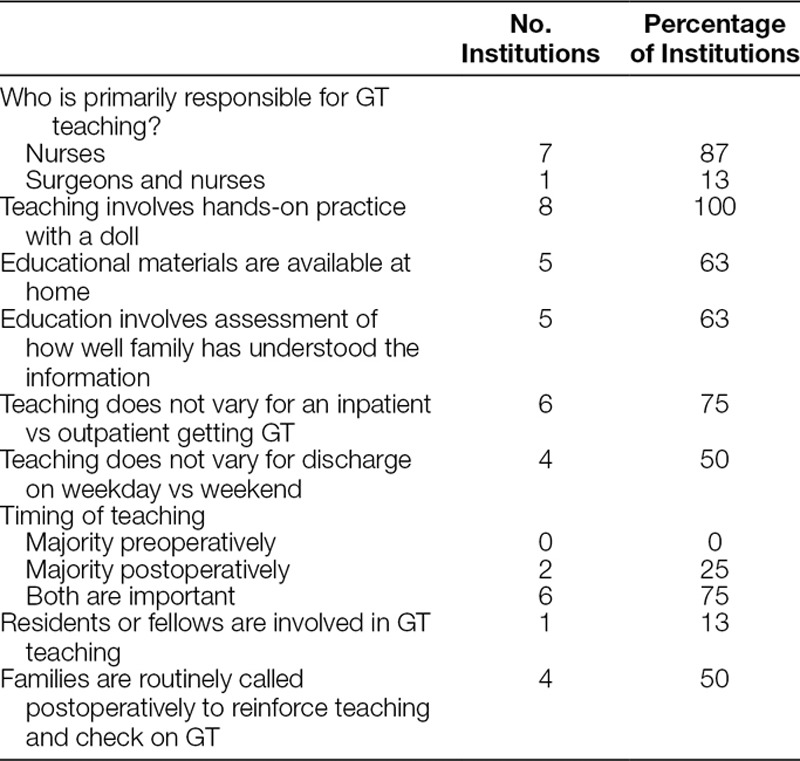
Quantitative Responses to GT Education Questions

Content presented during educational sessions varied, though all sites covered basic tube care and troubleshooting. All sites reported using a doll to practice hands-on tube placement and removal and familiarize families with general concepts of tube care. Two sites reported the use of an educational checklist. Specific educational topics were described as “tube dislodgement,” “what to expect,” “what is normal,” “how to manage complications,” “granulation,” “G tube care,” and “skin care.”

There were 3 main venues for GT education: the preoperative clinic visit, immediately after surgery while admitted to the hospital, and at subsequent postoperative clinic visits. Education was provided both in the preoperative and postoperative setting at 6 sites, whereas 2 sites primarily provided education postoperatively. Residents and fellows are not routinely involved in the education of patients and families in 7 sites.

Variation in education when there is a weekend discharge was reported at only 2 sites. An NP participant stated that “bedside nurses do the education at discharge. This can result in a difference in the quality of the teaching.” Six sites reported no variation in the education process. A surgeon participant reported that they “only schedule elective GTs Monday through Thursday, and avoid doing them on Fridays. Most GT stays are one night only.” One other site also reported that they plan the procedure timing to avoid a weekend discharge, and another reported that discharge is held until education is completed: “Nurses communicate how the teaching went with the team and request more time in the hospital occasionally.”

Only 1 site described a formal plan for education of alternate care providers that might not be present at preoperative or postoperative GT teaching sessions. This site provides educational classes for care providers twice per month in the clinic. The remaining hospitals indicated that whoever is present is taught or that they may come to the clinic for education if necessary. Interpreters are provided for educational sessions at all 8 sites, and 2 sites reported that educational materials are available in Spanish.

The 18 participants were asked to identify a single most important educational aspect that prevents visits to the ED or readmission. Two sites focused on setting realistic expectations: “Reinforce expectations: majority of tubes leak. Need wound care.” One nurse highlighted the importance of availability of expert support by phone: “If anything happens to the tube, then call.” Three sites emphasized the ability of the patient’s family to manage complications without going to the ED, as demonstrated by these quotes from nurse participants:

Focusing on self reliance, not an emergency, don’t need to come to ED unless THIS happens.

Post-op visit with ARNP: prophylactic steroid cream script, show pictures of granulation tissue. This keeps families out of ED. Also guidance about crusting and draining (Compare to ear piercing). Reassure them this is normal. Give specific parameters to keep easy skin things out of ED.

Know what to expect. Families should know how to troubleshoot and providers should be available.

Set realistic expectations. It will drain. Good wound care leads to success.

Written policy in handout - if anything happens to tube in first couple weeks then call directly rather than go to local ED.

### GT Technique

The surgeons interviewed at all 8 sites said that the vast majority of GTs are performed laparoscopically, though open and percutaneous endoscopic gastrostomy are utilized in rare specific cases (Table 3). Interventional radiologists rarely placed GTs but were often used to replace tubes or confirm replacement.

**Table 3.**
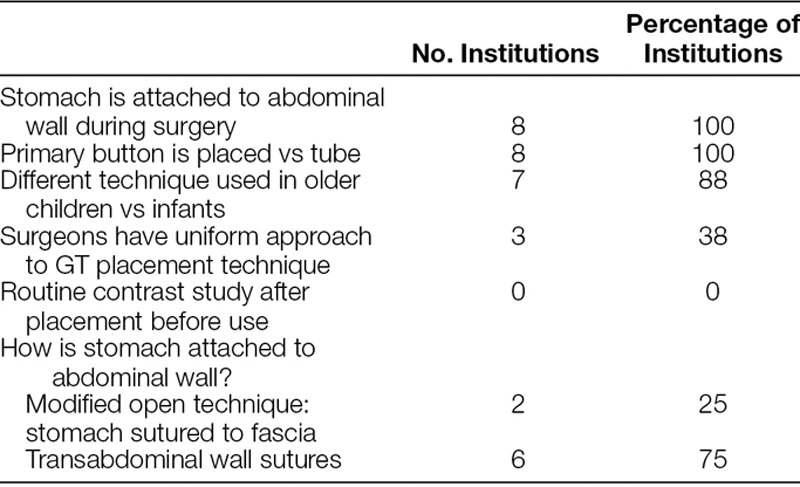
Quantitative Responses to GT Technique Questions

With regard to surgical technique, 3 of 8 centers have a uniform approach to GT placement. In the other 5 centers, surgeons noted that there was significant variability in technique among different surgeons. In all centers, the surgeons stated that the stomach was directly attached to the patient’s abdominal wall during GT placement. There were 2 main approaches to this: (1) direct placement of GT into stomach via gastrostomy and suture through stab incision to posterior rectus sheath (modified open technique—used primarily at 2 centers) and (2) Seldinger technique and sequential dilation to place GT and then securing stomach with transabdominal wall sutures (used primarily at 6 centers), which would be removed postoperatively or buried subcutaneously at time of surgery. Most of the centers using transabdominal wall sutures buried them subcutaneously. If they were not buried, then they were removed postoperatively as early as 2–3 days but up to 7 days after surgery. Half of the participants stated that the method of attaching the stomach to the abdominal wall did not affect outcomes. For those who felt that it did affect outcomes, some stated that the transabdominal wall sutures can cause irritation if left in too long and/or suture granulomas if buried. Proponents of transabdominal wall sutures felt the modified open technique resulted in wound breakdown and more site problems.

All sites used primary buttons for enteral access, but only 2 of the 8 surgeons said that the type of device used affected outcomes. One of these 2 stated that using a primary button as opposed to a Mic tube (Halyard Health, Alpharetta, GA) resulted in a lower rate of accidental dislodgement. Most surgeons did not secure the GT connection tubing to the abdominal wall at time of placement. Those that did used tape to secure this tubing to prevent jostling at the site and felt that this was important in minimizing chance of leak and site irritation.

Surgeons were more likely to perform PEGs or pure laparoscopic GTs with dilators in older children. Only 1 center did not vary its approach in older versus younger children. Overall, less than half of the surgeons interviewed (3/8) felt that the operative technique influences the likelihood of postoperative complications. Those surgeons who did think that operative technique was important described their rationale as follows:

Stitch that we place between the stomach and abdominal wall can get infected, but it may prevent dislodgement and need for OR return.

Careful technique - don’t pull stomach up into trocar wound in babies. Prevent mucosal prolapse. Anchoring stitch in posterior sheath and also anterior rectus sheath stitch placed prior to placement of g tube to get stomach under the rectus (anti prolapse stitch).

More leaks and granulation tissue when the tube is placed into the stomach through purse string versus just needle and dilator. Surgeon who did it that way retired. Another switched technique.

### GT Care

We asked participants whether GT patients are routinely evaluated by gastroenterology or any other services pre- or postoperatively, and all 8 hospitals said there was no routine care by services outside of the service placing the tube (Table 4). One center has an enteral access team, which is a multidisciplinary team involving surgical nurses and dieticians that is supposed to follow all GT patients, but in practice this does not always happen. Three other centers have a nurse-based team that sees GT patients regularly, but the other 5 centers stated that these patients are followed up by the team that places the tube. One nurse emphasized the importance of consistent care from the team who placed the tube: “Standardized approach is widely distributed and known throughout hospital. No random geneticist or neurologist giving advice (e.g., overinflate balloon). Parents want one place to go, one standard approach.” An NP participant reported that they had standardized the management of GTs “due to caregiver complaints about variation in processes.”

**Table 4.**
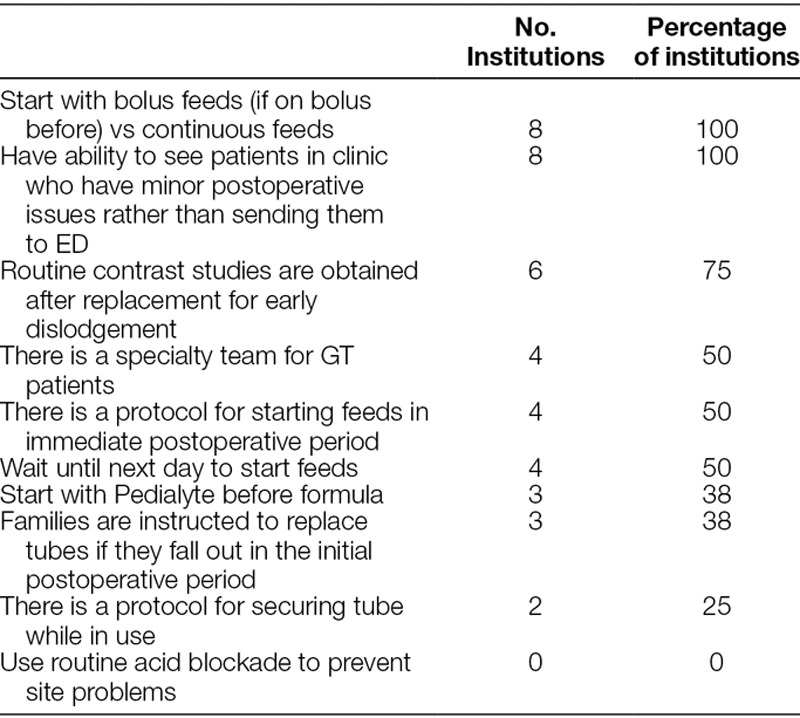
Quantitative Responses Regarding Approach to GT Care

With regard to immediate postoperative care, 4 of the 8 centers have a protocol that is followed while others vary according to the operating surgeon. There was some disagreement between surgeons and RNs at the same centers with regard to the existence of a protocol, with the RN being more likely to describe variation in practice according to surgeon preference. Overall, half of the centers tend to start feeds the day after surgery. The other half start sooner, with the shortest interval between end of surgery and start of feeds being only 4 hours. All centers start with bolus feeds if that is what the patient was on preoperatively, though they would usually start with half or less of the usual bolus amount. Five of the 8 centers start with formula, and the other 3 use Pedialyte (Abbott Laboratories, Abbott Park, IL) and then transition to formula.

Only 2 of the 8 centers reported that they have a protocol for securing the tube while in use, though there was again some disagreement between surgeons and nurses on this topic. Those who did have a protocol felt this was an important aspect of postoperative care to minimize motion at the site of the tube and resulting leak. One nurse emphasized the importance of communicating this concept to families: “We make a strong and strident case to parents about how delicate the healing gastrostomy is. Protect from accidental traction. That is part of discharge planning.”

We asked participants how they deal with early unintentional dislodgement of GTs. Participants defined the early period to be somewhere between 4 weeks and 3 months postoperatively, with most centers being closer to the 3-month mark. Three of the centers instruct families to replace the tube immediately if it becomes dislodged in the early period and then come to hospital for verification that the tube is in the stomach. The other 5 centers do not have families replace the tube in the early period but instruct families to come in for tube replacement. Almost all centers routinely obtain a GT study to confirm placement after early dislodgement. All 8 centers have clinic hours on all weekdays for patients to come in with GT problems, and patients are able to avoid ED visits by utilizing this option.

With regard to management of common site problems, participants described their approach to granulation tissue with a combination of topical steroid, silver nitrate, and operating room excision. Almost all participants stated that GT site infections are exceedingly rare and are usually much more likely to be irritation and improve with good site management rather than systemic antibiotics. A few centers use acid blockade in select cases to help with GT leak, but most approach this problem by excluding gastric outlet obstruction related to balloon, injecting more water in the balloon, and stabilizing or buttressing the tube at the site.

We asked participants about the most important aspect of GT care that results in prevention of readmission or ED visits. The following quotes are representative of the general nature of responses: “availability of specialty nurses,” “phone availability,” “extensive teaching,” “standardized approach,” “knowing when it is a true emergency,” “person to person teaching,” “troubleshooting,” “having everybody on board,” “setting realistic expectations,” and “having a number to call.”

Questions about the preoperative workup for patients undergoing GT placement and how patients are selected were beyond the scope of content covered in the interview. One participant did emphasize that this step is crucial in optimizing outcomes: “You know the old adage that says: ‘the best outcome from surgery is often from the operation you did not have to perform that day.’ I think we get wrangled into doing gastrostomies all too often by our colleagues. I wonder if the best outcome centers also make sure that our little patients really need the operation before they proceed….”

### Summary of Findings

**Fig. 1.**
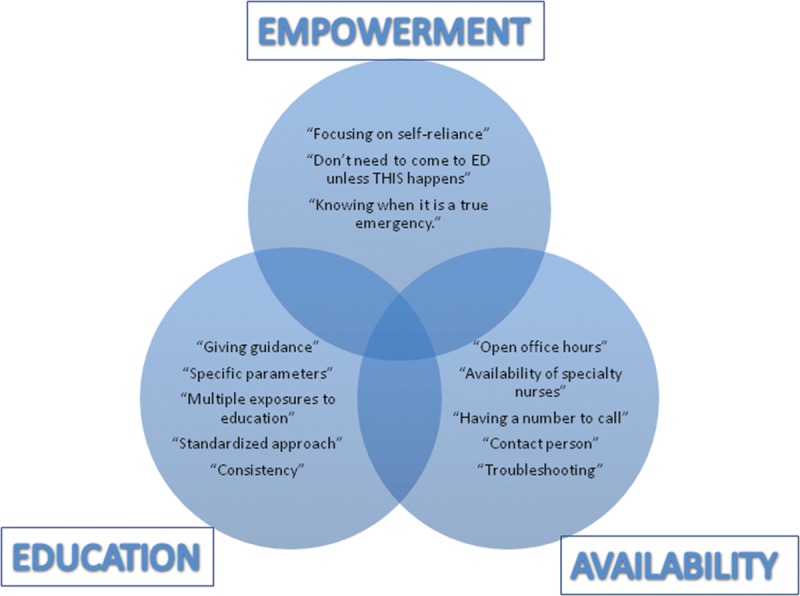
Themes from qualitative data analysis.

Overall, these findings can be distilled into 3 key concepts (Fig. 1):

1) EDUCATION: Teach families what to expect and set realistic expectations with continued reinforcement of these concepts.
2) AVAILABILITY: Be available by phone and in person to provide families with support and address concerns and minimize visits to the emergency room.
3) EMPOWERMENT: Reassure families they are capable of handling most minor issues that will arise.

In general, the participants expressed that technical aspects of operative placement and postoperative management of feedings and common complications are NOT as important as education, availability, and empowerment.

## DISCUSSION

A recent cohort study of children undergoing GT placement at children’s hospitals demonstrated that postoperative complications are common and resource intense, with over 8% of patients requiring at least 1 ED visit and nearly 4% of patients requiring readmission.^2^ Of the 38 hospitals evaluated, 8 were identified as low outliers for either 30- or 90-day ED revisits or readmissions. After interviewing surgeons and nurses from these institutions, we identified 3 common themes: caregiver education, availability of the healthcare team, and empowerment of the caregivers. Despite a plethora of research dedicated to evaluation of GT placement techniques,^12,13^ most participants did not emphasize technique as the aspect of care most linked to good outcomes; furthermore, surgical placement was not standardized at the majority of sites. It should be noted, however, that 100% of the centers interviewed stated that surgeons attach the stomach to the abdominal wall during the procedure and that surgeons place primary buttons as opposed to a GT that is later converted to a button. Apart from these 2 specific aspects of technique, there was a lot of variation within and across sites in terms of the technical approach to GT placement. Nuances in terms of tube selection, securing methods, and postoperative management were also not identified as key aspects to providing high-quality care.

All sites affirmed that education in the preoperative or immediate postoperative period was a critical aspect of high-quality GT care. Practice patterns such as avoiding elective GT placements on the weekends were reported to facilitate education. The paramount responsibility of GT education was under the purview of dedicated GT nurses at all centers. The practice of having a core group of educators perform GT teaching not only aids parents and caregivers at home but also informs and educates nurses and other healthcare providers.^14,15^ To this end, trainees in the form of residents and fellows were rarely used in education programs at these centers. GT educational programs not only improve outcomes such as revisits and readmissions but also improve caregiver comfort and mastery of skills needed to care for the GT and overall caregiver satisfaction. Details regarding the components of the educational process are beyond the scope of our review but included use of a model/doll, use of interpreters, and assessing comprehension.^16^ These may be particularly important to apply to patients and families for whom English is a nonnative language or for those who are otherwise challenged in their ability to process medical information.

The second theme was having experienced team members readily available to triage GT issues. Almost all sites provided a phone number for patients and caregivers to call. Telephone availability with 24-hour access to primary care physicians has been shown to reduce avoidable ED use from 41% to 8% of visits.^17^ Furthermore, availability of walk-in clinics that did not utilize the ED was a common resource available at the interviewed centers. Most of these clinics were colocated in proximity to the main hospitals where surgeons could walk over for quick assessment when needed. This may not be feasible for many centers with satellite or off-site clinics. Similar findings have been demonstrated in the primary care setting where availability of weekend clinic hours was associated with a 20% lower ED utilization compared with practices that did not have weekend hours.^18^

The final theme that emerged was empowering care providers so that they felt capable of managing the GT. This overlaps with providing a high level of education and assessing if the care providers are assimilating the information provided.^16^ Anderson and Funnell^19^ describe empowerment as “a process when the purpose of an educational intervention is to increase one’s ability to think critically and act autonomously.” Providing resources such as frequently asked questions documents, commonly encountered problems, and understanding when to call or seek additional medical attention are key aspects of empowerment. Spending time with care providers asking them to envision and talk through their action plans for common issues such as dislodgement or noticing granulation tissue can be helpful. A key aspect of caregiver empowerment assessment may be with review of discharge instructions, whereby a team member can reengage in education and assure the care providers that they have the knowledge and skills to provide GT care. Clear timelines and details regarding follow-up appointments can also empower care providers.^20^ Ultimately, families of patients with a GT are embarking upon a long-term relationship with the healthcare system and therefore these families should be empowered to optimize access to the system and understand how to connect with the healthcare providers available to them.

There are several limitations to this study. First, this is purely a descriptive study and we have not shown causality. We set out to describe practices in high-performing centers, but we have not proven that superior outcomes can be attributed to specific practices discussed in the interviews. In addition, we were unable to measure the consistency with which any individual high-performing center’s practice paradigms actually “reached” individual patients; some identified practices may have been intended but not executed.

Second, the centers interviewed were identified based on their revisit and readmission rates. There are clinically valid outcomes of interest such as dislodgement rates, patient satisfaction scores, and other unmeasured metrics that may have identified a different cohort of hospitals to qualitatively assess. Nonetheless, we assume that revisit and readmissions are valid quality measures that would be associated with additional favorable outcomes of interest.

Third, we did not interview low-performing centers to compare practices between high and low performers. Therefore, it is plausible that a center with poor outcomes may already provide care that is congruent with the themes and practices outlined in our study. Interviews with low performers would likely yield useful information about practices that may be associated with higher than average hospital revisits and readmissions. The limited number of centers interviewed is balanced by the depth of the interviews and the rigor with which the interviews and qualitative results were interpreted. It is precisely the qualitative nature of the analysis that brings strength to the conclusions by offering the reader in-depth commentary and themes common to the high-performing hospitals whose results are associated with these themes. In addition, the open-ended nature of qualitative responses allowed for generation of ideas that might not have been considered by the research team using traditional quantitative methods.

Fourth, the hospitals that comprise PHIS are typically those that see complex patients and tend to have extensive resources that can be used to provide care for patients with gastrostomies. Thus, the results of this study may not be generalizable to all hospitals that provide pediatric surgical care. Although this study identified various potential areas of quality improvement aimed at reducing unnecessary hospital revisits and readmissions, it is unlikely that one set of recommendations will fit all hospital systems and potential GT complications. As we learn more about process improvement in this particular area of pediatric surgical care, we are likely to find that the specific strategies needed to reduce ED revisits, for example, are different from those aimed at preventing hospital readmissions within a 90-day postoperative periods. Similarly, what works in a large tertiary care pediatric hospital with a small urban catchment area will likely differ from that in a smaller hospital with a wider rural catchment area.

Lastly, there are additional perspectives including the patient/parent point of view that are not reflected in this study that would be of great value and could be the focus of future qualitative studies evaluating GT care.

In conclusion, we have identified common themes among centers with favorable outcomes after pediatric GT placement. These themes included caregiver education, availability of the healthcare team, and empowerment of the caregivers. Technical aspects of GT placement and postoperative management seem to be less important. Identifying which components of GT care are associated with optimal outcomes is critical to our understanding of current practice and may help identify opportunities to improve care quality.

## ACKNOWLEDGMENTS

We gratefully acknowledge the Children’s Hospital Association for its logistical and administrative support throughout this project and Hannah Brown for her assistance in manuscript preparation.

## DISCLOSURE

The authors have no financial interest to declare in relation to the content of this article. This study was supported by departmental resources.

## APPENDIX

Interview guide: Identifying best practices for gastrostomy placement and postoperative care.

We recently completed a study showing that GT placement is associated with high rates of readmission and ED visits. We are interviewing you to better understand how the approach to GT placement and postoperative care may influence outcomes. We would like to ask you some questions about how GT patients are managed at your institution to identify best practices.

### Education (Ask NP or Other Dedicated GT Care Provider at the Institution)

Let’s talk about the general approach to gastrostomy teaching at your institution.

Who is primarily responsible for g tube teaching? What is the content of the teaching? Are materials available (online/handout/video) for the families at home? Is anything done to assess how well the family has understood the information? How does teaching vary if inpatient vs outpatient getting g tube? When is teaching done? (pre-op, post-op, both, etc) Does this vary if a patient is discharged on a weekend? Are residents or fellows involved in the teaching routinely? If so, how are they trained? Does anyone routinely call or contact the families postoperatively to reinforce teaching and assess GT status? What do you think is **the single most important educational aspect** at your hospital that prevents ED visits and readmissions?

### Technique (Ask Surgeon)

Let’s talk about the general approach to operative technique at your institution.

What techniques are used at your hospital (i.e. PEG, Lap, Open)? Which is the most common? Does IR do g tubes at your institution? Does GI do g tubes at your institution?

The remainder of questions refer to surgical GTs:

Is the stomach attached to the abdominal wall during surgery?

How is the stomach attached to the abdominal wall? Do you use trans-abdominal wall sutures? How long do they stay in post-op? Do you think the method of attaching stomach to abdominal wall influences outcomes? If so, how?

What type of device is most commonly used?

Button vs tube? Brand?

Do you think the type of device that is placed influences outcomes? If so, how?

Is the tube secured to the abdominal wall at the time of placement? How?

Do you think securing tube influences outcomes?

Do you use a different technique in older children (teenagers) vs. infants or younger children?

Do you routinely obtain radiographic gastrostomy studies to verify placement postoperatively before use?

Is there variation in surgical technique between surgeons within your organization?

Do you think the operative technique for g tube placement influences likelihood of post-op issues needing ER visit or readmission? Describe how.

What you think is **the single most important technical aspect of gastrostomy placement** that prevents complications at your institution?

### Postoperative Care and Inpatient/Outpatient Transition (Ask Surgeon and Advanced Practice Nurse)

Do other services such as gastroenterology routinely see the patients pre-operatively?

Do other services such as gastroenterology routinely see the patients post-operatively?

Do you have a specialty team for gastrostomies?

Protocol for starting feeds in immediate post-op period?

Time frame? Bolus vs continuous? Pedialyte vs. formula?

Protocol for securing tube while in use?

Is this important? How is it done?

Protocol for dealing with dislodgment of the gastrostomy, either early or late?

Define early/late. Families replace vs come to hospital? Routine g tube study after replacement?

Are there routine postoperative visits at a fixed interval, for example for gastrostomy change at 2 months or some other time?

Is the gastrostomy routinely changed at certain intervals or some set time?

How do you help parents stay out of the ED?

Who do the families contact when there is a problem, either during “busine hours” or in the evenings or weekends? When families do call with a problem, who determines whether they should stay at home/ come to clinic/come to ED? Do you have the availability to see patients in clinic who have minor post-op issues rather than sending them to ED? Are there any other systems are in place at your institution to minimize readmission/ER visits?

We would like to understand how you manage common g tube site problems at your institution.

How do you define and manage site infections? How do you manage granulation tissue? How do you manage leaking from the g tube? Is routine acid blockade used to prevent problems with the site?

**What do you think is the single most important aspect of gastrostomy care at your hospital that prevents readmission and/or emergency department visits?**

**Is there anything we have not already discussed that you think contributes to your good outcomes?**

**Is there anyone else at your institution we should consider talking to in order to better understand what results in good or bad g tube outcomes?**
